# The crystal structure of *Haloferax volcanii *proliferating cell nuclear antigen reveals unique surface charge characteristics due to halophilic adaptation

**DOI:** 10.1186/1472-6807-9-55

**Published:** 2009-08-22

**Authors:** Jody A Winter, Panayiotis Christofi, Shaun Morroll, Karen A Bunting

**Affiliations:** 1 Institute of Genetics, University of Nottingham, Queen's Medical Centre, Nottingham, NG7 2UH, UK; 2 Current address: Centre for Biomolecular Sciences, University of Nottingham, University Park, Nottingham, NG7 2RD, UK

## Abstract

**Background:**

The high intracellular salt concentration required to maintain a halophilic lifestyle poses challenges to haloarchaeal proteins that must stay soluble, stable and functional in this extreme environment. Proliferating cell nuclear antigen (PCNA) is a fundamental protein involved in maintaining genome integrity, with roles in both DNA replication and repair. To investigate the halophilic adaptation of such a key protein we have crystallised and solved the structure of *Haloferax volcanii *PCNA (*Hv*PCNA) to a resolution of 2.0 Å.

**Results:**

The overall architecture of *Hv*PCNA is very similar to other known PCNAs, which are highly structurally conserved. Three commonly observed adaptations in halophilic proteins are higher surface acidity, bound ions and increased numbers of intermolecular ion pairs (in oligomeric proteins). *Hv*PCNA possesses the former two adaptations but not the latter, despite functioning as a homotrimer. Strikingly, the positive surface charge considered key to PCNA's role as a sliding clamp is dramatically reduced in the halophilic protein. Instead, bound cations within the solvation shell of *Hv*PCNA may permit sliding along negatively charged DNA by reducing electrostatic repulsion effects.

**Conclusion:**

The extent to which individual proteins adapt to halophilic conditions varies, presumably due to their diverse characteristics and roles within the cell. The number of ion pairs observed in the *Hv*PCNA monomer-monomer interface was unexpectedly low. This may reflect the fact that the trimer is intrinsically stable over a wide range of salt concentrations and therefore additional modifications for trimer maintenance in high salt conditions are not required. Halophilic proteins frequently bind anions and cations and in *Hv*PCNA cation binding may compensate for the remarkable reduction in positive charge in the pore region, to facilitate functional interactions with DNA. In this way, *Hv*PCNA may harness its environment as opposed to simply surviving in extreme halophilic conditions.

## Background

Analysis of the archaeal DNA replication and repair machinery has highlighted key similarities with eukaryal rather than bacterial processes and is generally regarded as a good model to understand replication and repair in eukaryotes [1]. One emergent model organism is the extreme halophilic euryarchaeon, *Haloferax volcanii*, which may be studied using a range of approaches, including genetics, proteomics, biochemistry and structural analysis [2]. In particular, the relative ease of genetic manipulation compared to other model archaea has consolidated its use in the laboratory [3].

Extreme halophiles do not simply tolerate high salt concentrations, but in fact require in excess of 1 M NaCl to support growth [4]. To combat the high level of osmotic stress this places them under, halophiles accumulate inorganic ions, K^+ ^and Cl^-^, at intracellular concentrations approaching saturation [5]. All cellular proteins have therefore adapted to function under these extreme conditions. Various strategies for the halophilic adaptation of proteins have been identified, including the accumulation of acidic residues at the protein surface, a highly ordered solvent network [6], counterbinding of ions [7] and an increase in ion pairs [8] but these are by no means universal [9,10]. An understanding of how proteins remain soluble and active under these conditions is of interest both in terms of understanding adaptation to extreme environments and in biotechnology, to enhance protein engineering strategies.

One key component of the DNA replication machinery that is conserved between archaea and eukaryotes is the proliferating cell nuclear antigen (PCNA) processivity factor. PCNA is a trimeric, ring-shaped molecule with pseudo-hexagonal symmetry that can accommodate double-stranded DNA through a central pore. PCNA and other processivity factors tether polymerases to DNA to increase their processivity and are commonly referred to as sliding clamps. Despite very limited sequence identity (as little as 10% with the dimeric bacterial equivalent, known as β-clamp) there is a very high degree of structural conservation from phage through to humans [11-14].

Sliding clamp processivity factors have been shown to be essential for replication and genome maintenance in all domains of life. Initial interest focused on their interactions with DNA polymerases and a conserved binding motif (PCNA-interacting peptide or PIP-box) was identified [15]. This motif facilitates binding of DNA polymerases to the sliding clamps, enabling the high speed, processive DNA synthesis required for genome replication. Subsequently, a wide range of DNA modifying and repair enzymes have been found to interact with processivity factors, reinforcing the role of processivity factors as essential organisational components of the replication machinery.

Each processivity factor monomer possesses one binding pocket for a PIP-box bearing protein. This means that the trimeric archaeal and eukaryotic PCNAs can bind up to three partners simultaneously and the dimeric bacterial β-clamps two [16,17]. In this fashion processivity factors are thought to facilitate hand-off between enzymes in related pathways, providing temporal and spatial organisation [18]. PIP-box motifs are normally located at the extreme N- or C-terminus of the binding partner and the overall nature of the interaction between PIP-box motif and processivity factor is conserved from phage through to humans [17]. The PIP-box consensus motif has been defined as QxxI/L/MxxFF/Y [15]. The conserved glutamine residue forms direct and water-mediated hydrogen bonds with the surface of PCNA, whilst the aromatic components dock into a hydrophobic pocket situated near the interdomain connector loop [12]. Given both the conserved nature of this interaction and the vast number of identified binding partners, recent interest has focused on establishing how access to PCNA, and therefore the DNA substrate, is regulated [19].

How has PCNA with such strict structural conservation adapted to be soluble and functional at high salt concentrations and maintain its two key attributes under such conditions, as a sliding clamp on DNA and as a binding platform for numerous partners? To investigate these questions we have over-expressed and purified the *H. volcanii *PCNA (*Hv*PCNA), and solved the crystal structure to 2.0 Å, sufficient resolution to visualise solvent components at the protein surface.

## Results

### Overall architecture

**Figure 1:**
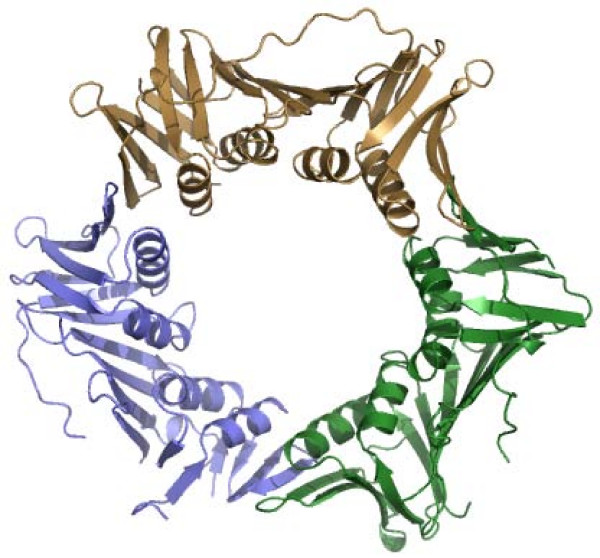
**Architecture of *Hv*PCNA**. Cartoon representation of the *Hv*PCNA trimer. Chain A is shown in purple, chain B in green and chain C in gold.

The crystal structure of *Hv*PCNA was solved to 2.0 Å resolution and contains one trimer in the asymmetric unit. The overall architecture is very similar to other known PCNA structures, as expected (Figure 1). Each domain contributes two α-helices to line the ring, supported at the outer edge by two β-sheets. Regions where disorder precluded modelling with any degree of confidence were excluded from the final model. These are: chain A residues 29–31, 60–64, 81–84, 90–96, 109–111 and 116–119 and chain B 91–95 and 124–126. Chain C is continuous except for residues 81 and 82. The N-terminal methionine is missing and the C-terminus is disordered in all chains and is not modelled from residue 244. Subsequent analysis will refer to chain C, as the electron density in this monomer is the most extensive and clearly defined. 274 water molecules and nine sodium ions are modelled in the asymmetric unit.

Comparative analysis is based on the known structures of PCNA, excluding those complexed with peptides or other binding partners. These are human (hPCNA [PDB:1VYM][20]), yeast (yPCNA [PDB:1PLQ][21]), *Pyrococcus furiosus *(*Pf*PCNA [PDB:1GE8][14]) and *Archaeoglobus fulgidus *(*Af*PCNA [PDB:1RWZ][18]). Calculated rms deviations range from 1.3 Å (*Pf*PCNA over 223 residues) to 1.7 Å (yPCNA over 225 residues), demonstrating the high degree of structural conservation within the PCNA family, despite their diverse archaeal and eukaryal origins.

### Monomer-monomer interface

Adaptation at subunit interfaces to enable archaeal multimeric proteins to remain stable under extreme conditions has been observed. In the tetrameric malate dehydrogenase of *Haloarcula marismortui*, intersubunit ion pair clusters have been identified in regions of the interface that are solvent exposed and presumably more sensitive to salt concentration [22]. Similar interactions were noted in the *Halobacterium salinarum *dodecin structure [23]. An increase has also been observed in *Pf*PCNA, which functions at high temperature [14].

**Figure 2:**
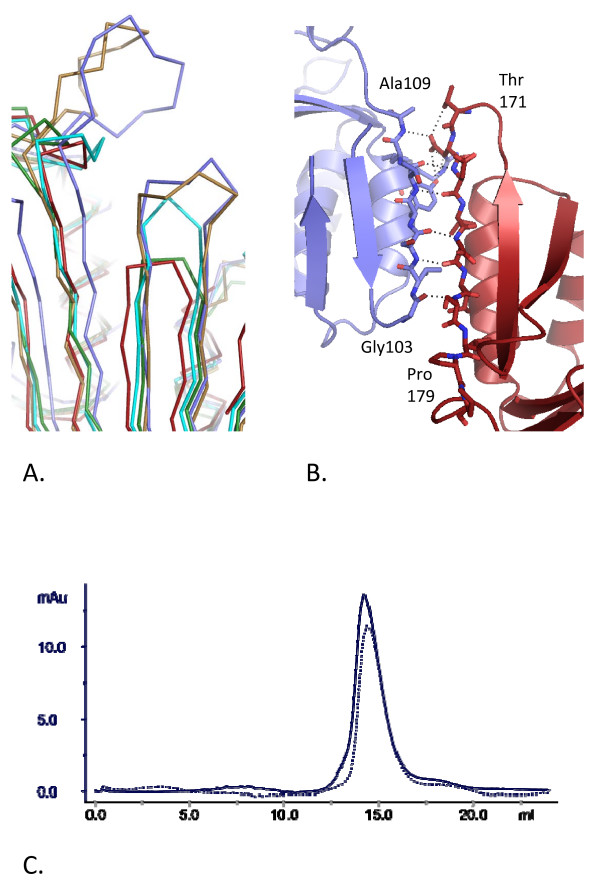
**Interactions at the monomer-monomer interface**. A. Superposition of known PCNA structures, showing variation in the extent of the monomer-monomer interface. Red – *H. volcanii*; green – *A. fulgidus *[PDB:1RWZ]; cyan – *P. furiosus *[PDB:1GE8]; purple – human [PDB:1VYM] and gold – yeast [PDB:1PLQ]. B. The monomers are coloured separately in red and purple, with individual side chains directly involved in interactions shown in stick representation. Residues at the end of the two β-strands involved are labelled. Hydrogen bonds are indicated by dashed lines. C. Size exclusion profiles of *Hv*PCNA in 0.2 M KCl (solid line) and 3.0 M KCl (dotted line). The *x *axis indicates elution volume (mls) and the *y *axis shows absorbance at 280 nm.

Whilst the architecture of the monomer-monomer interface is generally conserved in the known PCNA structures and principally involves antiparallel β-strands forming an extended β-sheet across the interface, the extent of hydrogen bonding and ion pairing between monomers varies. Analysis using the PISA server [24] shows that the buried surface area at the *Hv*PCNA interface is relatively small at 1160 Å^2 ^(averaged over the ncs related subunits). Only *Af*PCNA shows a smaller buried surface area of 1056 Å^2 ^(*Af*PCNA) compared to 1436 Å^2 ^(*Pf*PCNA), 1308 Å^2 ^(hPCNA, averaged over ncs-related subunits) and 1809 Å^2 ^(yPCNA). The shortening of two strands of the β-sheet on one side of the archaeal interface (residues 102–103, *Hv*PCNA numbering) restricts the potential number of main chain amide-carbonyl hydrogen bonds (Figure 2A), reducing the size of the monomer-monomer interfaces in the archaeal PCNAs, compared to the eukaryotic ones.

The hydrogen bonding interactions seen between *Hv*PCNA monomers are most similar to those of *Af*PCNA. At the *Af*PCNA interface, only six main chain hydrogen bonds and one involving side chains are formed, consistent with the reduction in the intersubunit β-sheet. *Hv*PCNA also has a relatively small interface and so forms just five main chain hydrogen bonds, with further side chain interactions involving Asp172 OD2 with Tyr106 OH and Ala109 N (Figure 2B, Additional File 1: Table S1). Also striking is the low number of ion pairs formed by *Hv*PCNA and *Af*PCNA, more reminiscent of the eukaryotic PCNAs than *Pf*PCNA. In yeast, human, *A. fulgidus *and *H. volcanii *PCNAs, one side chain from each monomer participates in ion pairing (Arg72 and Asp172 in *Hv*PCNA). In comparison, *Pf*PCNA forms 10 ion pairs at the interface [14].

Given the absence of additional ion pairs in *Hv*PCNA the stability of the trimer over a range of KCl concentrations (0.2 to 3.0 M) was assessed using size exclusion chromatography. Figure 2C shows the elution profiles in the extreme conditions 0.2 and 3.0 M KCl for clarity. In all KCl (and equivalent NaCl) conditions tested, *Hv*PCNA eluted at a position consistent with a molecular weight of 80 kDa (trimer) based on calibration with molecular weight standards. This analysis suggests that the *Hv*PCNA trimer is stable in both low and high salt conditions even after incubation for four hours.

### Surface charge and conserved residues

One of the most characteristic forms of halophilic adaptation is an increase in surface exposed acidic residues. It was of particular interest to analyse the charge distribution of *Hv*PCNA, given that a marked feature of the sliding clamps is an overall acidic character, except for the substantial electropositive potential lining the central pore and held to be essential for function [11,21]. Alteration in amino acid usage is a commonly reported feature of halophilic euryarchaeota, with an increased number of aspartate residues accompanied by a reduction in lysine most frequently observed [22] (Additional File 1: Table S2). The expected increase in aspartate usage (11.3%) is observed in *Hv*PCNA, although all of the PCNAs shown contain more aspartate than average, as expected in proteins of an acidic nature. Lysine usage is markedly reduced at 2.8% in *Hv*PCNA, compared to the next lowest (hPCNA) at 6.1% and this has significant consequences for the surface charge distribution. Alanine usage is high (11.3%) compared to the other PCNA structures (5.0–7.8%), also reported previously [6]. The net charge, calculated assuming histidines to be neutral, of *Hv*PCNA is -31, considerably higher than the remaining PCNA structures (-13 to -20) [25].

**Figure 3:**
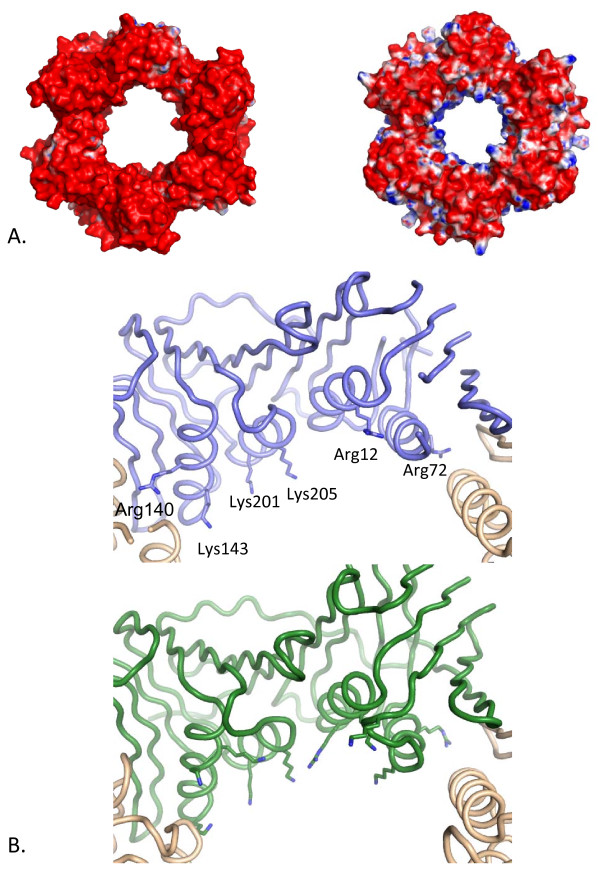
**Surface charge distribution of *Hv*PCNA compared with *Af*PCNA**. A. Electrostatic surfaces of *Hv *(left) and *Af*PCNA (right [PDB:1RWZ]) demonstrate that the acidic nature of PCNAs is more pronounced in *Hv*PCNA and that the halophilic protein lacks the positive electrostatic charge characteristic of the inner channel. The electrostatic potential was calculated using the APBS package [43]. The accessible surface area is coloured according to the calculated electrostatic potential from -10 k_B_T/e (red) to +10 k_B_T/e (blue). B. Penetration of basic residues into the central channel of *Hv*PCNA (top) and *Af*PCNA (bottom). The structures are depicted with a backbone trace with basic residues located on the α-helices lining the central pore depicted in stick representation. In *Hv*PCNA only Lys143 and Lys205 project into the channel in the manner seen in classical PCNAs. Arg12, Arg72 and Arg140 are involved in substantial interactions with protein atoms and Lys201 is involved in charge neutralisation at the sodium cluster site. In contrast the majority of the basic residues lining the *Af*PCNA pore project into the channel.

Whilst PCNA is known to be a predominantly acidic protein the inner pore usually bears a positive electrostatic charge, proposed to enable free movement along the negatively charged DNA backbone without repulsion [21]. The remaining protein surfaces are predominantly negatively charged (Figure 3). *Hv*PCNA is markedly different from typical sliding clamps, with almost total loss of the usual positive surface charge in the inner pore. The surface of the more typical *Af*PCNA is shown for comparison (Figure 3), since it has the highest sequence identity to *Hv*PCNA of the solved structures (36%), along with yeast, human and *Pf*PCNA (Additional File 1: Figure S1 and Additional Files 2 and 3).

The positive electrostatic surface in the pore region of typical PCNAs is due to an array of lysine and arginine residues on the 12 α-helices that line the pore. Classical PCNAs have 9–12 lysines and arginines in this region (per monomer), the majority projecting into the central pore. In stark contrast, as shown in Figure 3, there is very limited positive electrostatic surface lining the *Hv*PCNA pore. Examination of the inner ring reveals that *Hv*PCNA possesses only two basic residues (per monomer), Lys143 and Lys205, that extend into the channel and are likely to contribute significantly to the postulated water-mediated interactions with the phosphate backbone of DNA passing through the pore [11].

The remaining basic residues lining the *Hv*PCNA pore are involved in interactions with other protein atoms and, as such, are unlikely to mediate *Hv*PCNA-DNA interactions (Figure 3B). (i) Arg12 does not penetrate the central channel, instead forming hydrogen bonds with a solvent molecule and the main chain carbonyls of Ala77 and Ala80. (ii) Arg72 and Arg140 are located in the vicinity of the monomer-monomer interface and are directed away from the pore. In particular, Arg72 is substantially involved in interactions at the monomer-monomer interface, forming an ion pair with Asp172 in the opposing monomer. (iii) Lys201 is involved at the periphery of the sodium-binding cluster located around residue 150 (described below), forming ion pairs with Asp146 and Asp198.

### PIP-box binding

**Figure 4:**
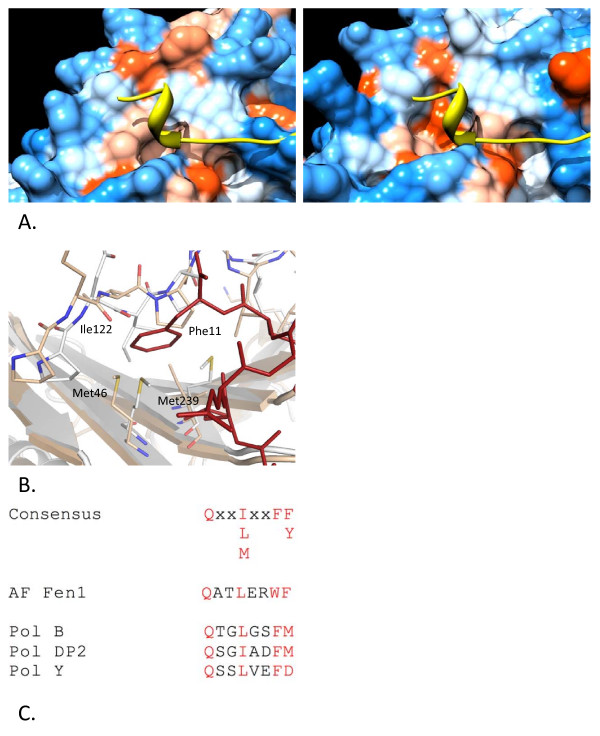
**Analysis of the hydrophobic PIP-box binding pocket on the surface of *Hv*PCNA**. A. Hydrophobic surface of *Hv*PCNA (left) and uncomplexed *Af*PCNA (right – [PDB:1RWZ];) with the backbone of the *Af*Fen1 peptide [PDB:1RXZ];shown in yellow. Amino acids are coloured according to the Kyte-Doolittle scale with blue for the most hydrophilic residues to white (0.0) and orange-red for the most hydrophobic. Produced using Chimera [44]. B. Superposition of *Hv*PCNA (white) and *Af*PCNA (beige) [PDB:1RWZ] with the Fen1 peptide depicted in red (from [PDB:1RXZ];. Met46 and Met239 and interdomain connector loop residues are shown in stick representation with atomic colouring (*Hv*PCNA numbering). C. Alignment of the candidate PIP-boxes of *H. volcanii *DNA polymerases with the PIP-box consensus sequence and that of *Af*Fen1 [PDB:1RWZ]. Conserved residues are highlighted in red.

Comparison of the PIP-box binding surface of *Hv*PCNA with *Af*PCNA suggests this region is generally well conserved. However, Met46 in *Hv*PCNA has shifted in orientation compared to both the unbound and complexed forms of *Af*PCNA. This increases the accessible surface area to 37.0 Å^2 ^in *Hv*PCNA compared to 23.1 Å^2 ^in *Af*PCNA. This position is occupied by leucine in the remaining PCNA structures and the orientation of the side chain is more similar to *Af*PCNA than *Hv*PCNA. The unusual orientation of Met46 in *Hv*PCNA, combined with the substitution of Met at position 239 makes the binding pocket for the turn of 3_10 _helix in the PIP-box shallower and less hydrophobic in nature (Figure 4A). Slight deviations in the interdomain connector loop (residues 109–129) have caused Ile122 to shift slightly in position, further restricting the binding pocket (Figure 4B).

The C-terminal regions of the *H. volcanii *replicative polymerases, PolB and PolD2 and the Y-family translesion polymerase, PolY, were analysed. All three have candidate PIP-box motifs in good agreement with the consensus sequence (Figure 4C). The only significant deviation is in the terminal residue of PolY. This is aspartate, in stark contrast to the bulky aromatic group normally found in this position. The 3_10 _helix in the PIP-box motif characteristically inserts two large aromatic groups into the hydrophobic pocket at the base of the interdomain connector loop and this substantial hydrophobic interaction plays a key role in binding. Modelling the sequence of the putative PIP-box of PolY onto the *Af*Fen1 peptide suggests a close, unfavourable contact between the PolY C-terminal Asp and Met46 of PCNA. The equivalent residue in the replicative polymerase PIP-boxes is methionine which, given the relative flexibility of its side chain, presumably could be accommodated in the binding pocket.

### Solvent network and crystal packing

Solvent molecules were introduced during refinement and rebuilding according to the approach described by Richard and others [8]. More water molecules were assigned to chain C than to chains A or B, reflecting the more defined electron density in this region (Additional File 1: Table S3). Whilst the number of solvent molecules identified in the known PCNA structures is highly variable and difficult to compare objectively as criteria for retention vary, it is apparent that the water molecules in *Hv*PCNA are very well defined and refine with a markedly lower temperature factor than those in the remaining structures. This is suggestive of a highly ordered solvent network, consistent with findings in other halophilic proteins [6]. The direct protein contacts involved in crystal packing in *Hv*PCNA are limited and predominantly involve ion pairs between arginine and aspartate/glutamate residues, totalling nine across the trimer. One main chain hydrogen bond occurs between Pro41O-Gln118N in chain C with the OE1 group of the glutamine forming an additional bond with the ND2 group of Asn43. The carbonyl oxygen of Glu93(C) forms a hydrogen bond with the NH2 group of Arg37(B). Other contacts are water mediated, with the largest interface located around the two-fold axis and centred on the carbonyl group of Leu183. This interface involves substantial networks of water molecules, as has been noted in previous structures of halophilic proteins [6,25]. In addition, analysis of one interface suggested the presence of a non-water solvent ion and this is discussed in more detail below.

### Cation binding

Another approach postulated for halophilic adaptation of proteins is the presence of bound anions and cations [22], observed in many of the structures solved to date of sufficient resolution [6,8,23,25]. Whilst all solvent molecules were originally assigned as waters, these were investigated further if they possessed more than four hydrogen-bonding partners, distances lower than normal for hydrogen bonds and low refined temperature factors. Such molecules were initially modelled as Na^+^, since this ion was abundant in the crystallisation liquor and examination of peaks in the *Fo-Fc *maps were not suggestive of Ca^2+^, also present. Following careful refinement, three Na^+ ^ions over two sites were retained in each monomer.

**Figure 5:**
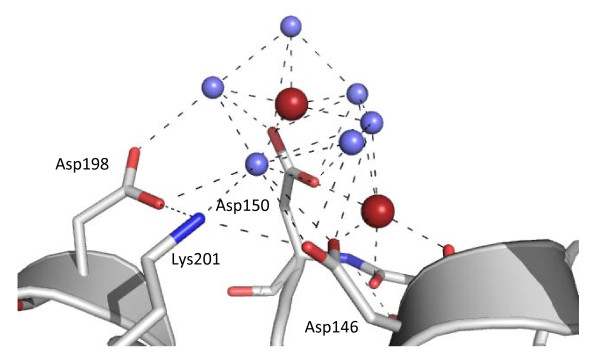
**The sodium cluster adjacent to Asp150**. Water molecules are shown in purple and sodium ions in red with hydrogen bonds indicated by dashed black lines. Asp146, Asp150, Asp198 and Lys201 are labeled and shown in stick representation. The main chain carbonyl groups of Asp 146 and Ser149 are also shown.

The principle cation binding site is located around a carboxylate cluster (Asp146, Asp150 and Asp198), towards the inner edge of the ring and appears to contain two Na^+ ^ions coordinated by the carboxylate groups, water molecules and main chain carbonyls (Figure 5). One sodium ion is coordinated with tetragonal bipyramid geometry by the side chains of Asp146 and Asp150, carbonyl groups of Ser149 and Asp150 and one solvent molecule. One solvent molecule is absent and is presumably more mobile due to the lack of constraints from forming additional hydrogen bonds with protein atoms. The other sodium ion is coordinated by both OD1 and OD2 of Asp150 and by five solvent molecules in a slightly distorted planar pentagonal fashion. One of the solvent ligands is additionally associated with two solvent molecules and the OD1 groups of Asp146, Asp150 and Asp198. Interestingly the NZ group of Lys201 clearly interacts with Asp146 OD2 and Asp98 OD1, its associated charge permitting the acidic side chains to orientate in this fashion.

A second site is located at a crystal-packing interface between Leu127 and Asp157 in the neighbouring monomer (Additional File 1: Figure S2). The carbonyl oxygen of Leu127 interacts directly with the sodium ion, which is further coordinated by four water molecules involved in interactions with Asp157, Leu127, Ala129 and Glu216. Details of the interactions in each monomer are shown, together with average bond lengths (Additional File 1: Table S4 and S5).

## Discussion

The degree of structural conservation in processivity factors from phage through to humans is marked. The trimeric PCNAs and dimeric β-clamps presumably arose from a common, single domain ancestor by gene duplication and fusion events [26]. Further, the structures of halophilic proteins solved to date indicate that overall folds are generally well-conserved when compared with their non-halophilic counterparts. Adaptation to high salt conditions tends to involve changes to the surface exposed residues or addition of small structural elements (halophilic addition) rather than large structural changes [6,9,25,27]. Taking these considerations into account, it is not unexpected that the overall architecture of *Hv*PCNA is very similar to the known homotrimeric PCNA structures, both archaeal and eukaryal.

### Little evidence for halophilic adaptation is seen at the monomer-monomer interface

One common form of halophilic adaptation that is not seen in *Hv*PCNA is an increase in inter-subunit ion pairs, observed in a number of multimeric halophilic protein structures [8,23]. Clearly adaptation reflects the nature of interfaces and protein function, even within the individual protein; intersubunit ion pairing in the *H. marismortui *malate dehydrogenase is more extensive in the dimer-dimer than monomer-monomer interfaces, for example [8].

Unlike many proteins where the multimeric interfaces remain intact, the interface in PCNA is required to open for loading onto DNA. However, the formation of excess ion pairs in *Pf*PCNA does not prevent loading of this protein onto DNA [28]. Size exclusion chromatography shows that *Hv*PCNA remains trimeric under a wide range of KCl conditions. This suggests that the interface was already sufficiently stable at extreme salt concentrations to maintain the multimeric form and thus no selection pressure was brought to bear requiring this form of halophilic adaptation.

Although the overall architecture of the monomer-monomer interface is conserved, a smaller surface area is buried in the archaeal PCNAs as compared to the eukaryotic proteins. The consequent reduction in main chain hydrogen bonding interactions was noted in the *Pf*PCNA structure and was postulated to explain the ability of *Pf*PCNA to self load on circular DNA, in the absence of the RFC clamp loader [28,29]. It seems likely, given the further reduction in the buried surface area of *Hv *and *Af*PCNA, that such self-loading is common to archaeal PCNAs and may prove advantageous to organisms living under extreme conditions.

### Amino acid usage affects the characteristic electrostatic charge distribution of *Hv*PCNA

The most commonly observed form of halophilic adaptation, although by no means universal, is an increase in surface exposed acidic residues, coupled with a reduction in lysine usage [22,30]. These previously reported trends certainly hold true for *Hv*PCNA, as well as increased use of alanine, presumably related to the lower hydrophobicity of this amino acid [30]. The sliding clamps are all highly acidic proteins, but the effect is more exaggerated in *Hv*PCNA as can clearly be seen by mapping the electrostatic potential onto the accessible surfaces of *Hv *and *Af*PCNA (Figure 3).

The most unusual and striking feature of *Hv*PCNA is the almost complete absence of the typical distribution of positive electrostatic charge on the inner surface of the ring, usually provided by arginine and lysine side chains [11,21]. The few positively charged residues that are present are mostly involved in substantial interactions with other protein atoms. The positively charged pore lining is predicted to reduce repulsion effects with the negatively charged phosphate backbone of DNA, although interactions are likely to be water mediated if DNA passes perpendicularly through the clamps [11]. Recently it has been suggested that DNA may be tilted under certain circumstances and directly contact the PCNA inner ring. Single particle analysis of *Pf*PCNA implicated a number of residues in these contacts [31]. Of these, *Pf*Lys209 equates to one of the two lysines (Lys205) in *Hv*PCNA that do project into the channel and *Pf*His75 is equivalent to *Hv*Arg72. Whilst this residue does not protrude directly into the channel in *Hv*PCNA, interaction with tilted DNA is not precluded.

While the precise positions of the arginine/lysine residues are variable [32], the overall positive electrostatic potential lining the inner pore is conserved in all other sliding clamp structures to date. The *H. salinarum *nucleoside diphosphate kinase binds nucleotide in a virtually identical manner to a human homologue despite altered amino acid usage for halophilic adaptation [27] and conservation of residues involved in substrate recognition and binding has also been observed in other halophilic structures [6,9]. Presumably disfavoured residues are tolerated when they are required for protein function. It is interesting to note that a residue postulated to be involved in direct contact with DNA is one of the few positive residues retained in the *Hv*PCNA pore.

*H. volcanii *PCNA is generally accepted to be an essential protein in the cell [33]. It is most likely to function as a processivity factor, in which case the lack of a positive electrostatic potential does not abrogate its ability to slide on DNA *in vivo*. Mutational analysis of human PCNA involving the basic side chains lining the central channel suggested that although they were essential for stimulation of polymerase activity, the mutations affected efficient initiation, not actual clamp sliding along DNA [34]. Nonetheless, a single mutation in hPCNA had a severe effect on clamp function and, by comparison, the reduction in basic side chains in *Hv*PCNA is dramatic. How does *Hv*PCNA slide on DNA? One plausible explanation is that bound counterions may neutralise the electrostatic repulsion between the carboxylate side chains and the phosphate backbone (see below). In addition, the effect of bound water molecules lining the channel, first noticed in the β-clamp structure [11], may be more profound for *Hv*PCNA given the stability and extent of hydration shells in halophilic proteins [6].

### The *Hv*PCNA PIP-box pocket is shallower and less hydrophobic

The overall composition of the hydrophobic pocket involved in the interaction with PIP-box peptides is relatively well conserved in *Hv*PCNA. Modelling suggests that the overall mode of contact would be maintained. Putative PIP-box motifs in the sequences of *H. volcanii *replicative polymerases (PolB and PolD2) and a Y-family translesion polymerase, PolY (Figure 4C) all retain the conserved glutamine and a moderately hydrophobic residue in the central conserved position. The only deviation seen is in the bulky aromatic groups, typically FF or FY. Deviation in this position is not unknown – many thermophilic proteins, including the *A. fulgidus *Fen1 peptide bound in the 1rxz structure, possess tryptophan in this region [18,19].

The differences observed in the *Hv*PCNA structure are subtle and lead to a reduction but not elimination of the overall hydrophobic character, consistent with observations that halophilic proteins can demonstrate a reduced hydrophobic surface [6] (Figure 4). The depth of the hydrophobic pocket is reduced and it is likely that, in the intracellular environment of *H. volcanii*, a moderately hydrophobic residue such as methionine substituting for phenylalanine in the replicative polymerase PIP-boxes would permit stable binding, due to the exaggeration of hydrophobic effects in high salt conditions. The aspartate residue at the extreme C-terminus would not favourably interact with the hydrophobic pocket and given its terminal position could flip out of the pocket. Previous structural studies have suggested that Y-family polymerases PIP-boxes show a greater tendency to diverge from the canonical sequence than do replicative polymerases so this finding is not unexpected [17].

### The *Hv*PCNA solvent shell is well ordered and contributes to crystal packing

*Hv*PCNA possesses a well-ordered solvent network, extending into the second hydration shell. The majority of water molecules assigned in the *Hv*PCNA structure are in the first hydration shell and have temperature factors similar to the overall *B *factors for the protein atoms in each chain (Table 1). Networks of water molecules are observed at many of the crystal contacts with neighbouring molecules, a feature which has been observed in several crystal structures of halophilic proteins [6,25]. Utilisation of water-mediated crystal contacts has been postulated to compensate for the electrostatic repulsion effects of highly acidic molecules [25]. Ion pairs also play a role in crystal packing in *Hv*PCNA and are more evident than main chain and side chain hydrogen bonding interactions.

**Table 1:** Data collection and refinement statistics.

	All data (outer shell)
Data Collection	
	
Space group	*C*2
Cell dimensions	
a, b, c (Å)	83.4, 143.8, 78.0
α,β,γ (°)	90.0, 121.6, 90.0
Resolution (Å)	37.96-2.00 (2.11-2.00)
R_merge_	0.066 (0.315)
<I/σI>	11.6 (3.3)
Completeness (%)	95.3 (95.9)
Redundancy	2.5 (2.5)
	
Refinement	
	
Resolution (Å)	25.0-2.0
No. of unique reflections	47498
R_cryst_	0.214
R_free_	0.259
rms deviation from ideal values	
bond lengths (Å)	0.023
bond angles (°)	2.171
Number of atoms/au	5498
*B *factor (Å^2^)	
protein	
chain A	28.5
chain B	28.5
chain C	21.3
waters	27.6
Na^+ ^ions	22.0

### Cation binding

Surface-bound ions, both anions and cations, have been identified in the vast majority of halophilic protein structures [6,8,23,25]. Cations have been proposed to counter the charge effect resulting from the excess of acidic residues on the protein surface, although they have not been sufficiently evident in crystal structures to totally neutralise that effect [6]. Additionally, ion binding has been shown to be involved in substrate recognition [6] and in stabilising the complex salt bridges required to maintain the *H. marismortui *malate dehydrogenase tetramer [8].

Two ion-binding sites per monomer were observed in the *Hv*PCNA structure, comprising three ions in total. These were tentatively identified as sodium based on the bond lengths, lack of residual density following modelling water molecules and prevalence of Na^+ ^in the mother liquor. The sites are remarkably consistent throughout the ncs-related subunits.

One site is involved in crystal packing, a commonly observed feature [23,25]. It seems likely that many more cations are bound across the surface of acidic halophilic proteins and those at crystal packing interfaces are most easily visualised, due to their greater degree of order.

The sodium cluster located around Asp150 is considerably more intriguing. The carboxylate moieties face each other, which is not an energetically favourable conformation and is partially neutralized by the side chain of Lys201. Asp150 is conserved as an acidic amino acid, most usually aspartate, in all PCNA sequences currently available in SwissProt, with the exception of those from *Trichoplusia *and some viruses. Such conservation in a family of proteins that are notoriously poorly conserved at the sequence level hints at some currently unknown function that is perhaps protected by the sodium cluster.

**Figure 6:**
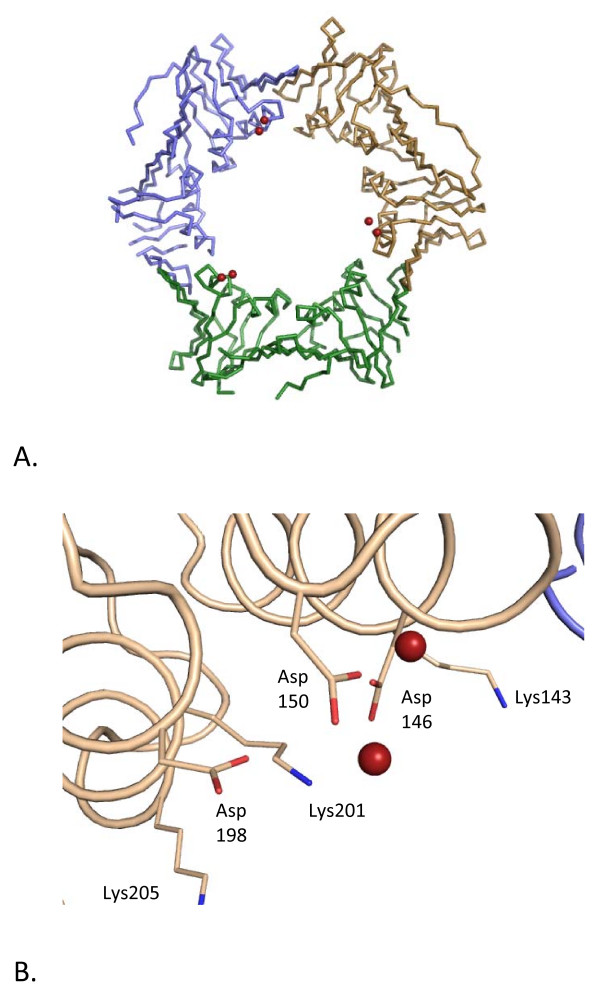
**Context of the sodium cluster in relation to the central pore**. A. Backbone representation of the trimer showing the global position of the cluster. Each monomer is coloured separately and the sodium ions within the cluster are shown as red spheres. B. The local environment of the cluster, demonstrating the proximity of the coordinating aspartates (Asp146, Asp150 and Asp198) to the three lysine residues within the channel (Lys143, Lys201 and Lys205). Lys201 is involved in charge neutralisation within the cluster. Sodium ions are shown as red spheres.

Another possibility is that the cations act to replace the basic residues that line the channels of other PCNAs (Figure 6). Although no further unambiguous cations have been identified lining the channel, previous studies have suggested proteins bind many more cations and waters than are visible utilising X-ray crystallography [8]. The cluster may provide a particularly stable conformation for cation binding, such that it is fully occupied and well ordered in the crystal. Sodium and water molecules are difficult to distinguish in electron density maps, particularly if occupancy is reduced by transient association. The pore is not accessible for crystal packing interactions, where order can reveal the presence of bound ions.

It seems likely that *Hv*PCNA contains many more surface bound cations than identified in this structure and that a proportion of these will line the central pore, given the presence of aspartate residues in this region. This is consistent with the proposal that ion-binding may not simply protect halophilic proteins but also allow them to harness their environment [8], in this case compensating for the loss of lysine residues and reducing charge repulsion effects between the acidic protein surface and phosphate backbone of DNA. Although the electropositive nature of the central pore is conserved amongst PCNAs the exact position of the residues is not. This lack of positional conservation may permit the basic side chains to be replaced by bound cations, except in positions where direct interactions with DNA may occur.

## Conclusion

The halophilic structures solved to date represent a diverse range of proteins and the adaptation each of these has undergone will reflect their own unique characteristics and functions within the cell. It is not surprising that, although trends exist, there is no single universal determinant for halophilic adaptation. *Hv*PCNA displays some common features and some unique facets. No increase in inter-subunit ions pairs is observed and this is consistent with the stability of the *Hv*PCNA trimer across a range of salt concentrations. Two common features are the increased acidity of the surface, despite the PCNA family already displaying marked electronegative surfaces, and the presence of bound ions, in this case cations, the latter potentially countering the effects of the former to maintain biological structure and function under extreme halophilic conditions.

## Methods

### Cloning, expression and purification

*Hv*PCNA was amplified from *H. volcanii *genomic DNA (wild type DS2[4]) and cloned into the *Nde*I/*Bam*HI sites of pACYC184-11b [35] for expression purposes. *Hv*PCNA was over-expressed in *E. coli *B834 (DE3); cells were grown in LB containing 34 μg/ml chloramphenicol at 37°C to an OD_600 _of 0.6–0.8. Expression was induced by the addition of 1 mM IPTG for 2 hours at 37°C. Cells were harvested by centrifugation and resuspended in buffer A (50 mM HEPES pH 7.0, 1.0 M NaCl) and protease inhibitor cocktail tablets (Roche) prior to lysis via sonication and clarification by centrifugation. The 60–80% fraction from ammonium sulphate precipitation was resuspended in buffer A and dialysed against buffer A prior to application to a 26/60 Superdex 200 (GE Healthcare) size exclusion column equilibrated and run in buffer A. A well-defined peak was observed at an elution volume consistent with that of an assembled trimer of PCNA. Fractions were pooled and diluted to a final concentration of 50 mM HEPES pH 7.0 and 400 mM NaCl for application to Q-sepharose FF (GE Healthcare) and were eluted in buffer A and concentrated using a Vivapore 10/20 7500 Da cutoff (Vivascience). Concentrated protein was stored at 4°C.

### Crystallisation and data collection

Crystals were grown using the sitting drop vapour diffusion method. 3 μl of *Hv*PCNA at 5 mg/ml was mixed with 3 μl of precipitant solution containing 0.2 M CaCl_2_, 0.1 M HEPES pH 7.5 and 18% PEG 400. The drops were equilibrated against 500 μl of the precipitant solution supplemented with 1 M NaCl and incubated at 10°C. Cubic crystals were obtained within two weeks and were washed in mother liquor and used for seeding fresh drops. This approach produced two crystal forms; form I crystals were cubic with dimensions of approximately 120 μm, form II crystals were smaller (50 μm and irregularly shaped) and were observed after several weeks and in a small proportion of wells. Form I crystals were cryoprotected via the addition of 0.6 μl glycerol and 0.7 μl PEG 400 to the drop and were allowed to dehydrate for 2 hours prior to freezing. Form II crystals were frozen directly in mother liquor. Data to 2.6 Å were collected on form I on ID14-4 at the ESRF and to 2.0 Å on form II at IO3 at the DLS.

### Structure determination/refinement

Data were processed using IMOSFLM and programmes contained in the CCP4 suite [36,37]. Molecular replacement on crystal form I was carried out using BALBES [38], with PDB:1RWZ as the search model, yielding a solution containing three monomers in the asymmetric unit. Visual inspection of the resulting maps confirmed the presence of a fourth monomer. The asymmetric unit contains one intact trimer, with the fourth subunit producing the biological trimer via the crystallographic 3-fold axis. The partially refined structure was used as the search model for the second crystal form, which diffracted to higher resolution. Molecular replacement in this instance was performed using MOLREP [39] and produced one trimer in the asymmetric unit. Refinement in both cases was performed using the twin refinement option in REFMAC [40] interspersed with manual building using the COOT visualisation package [41]. Water molecules and cations were assigned according to the criteria laid out by Richard and others [8]. No ncs restraints were applied during refinement. Data collection and refinement statistics are shown in Table 1. Figures were prepared with the PyMol molecular graphics package unless stated otherwise [42]. Coordinates have been deposited at the Protein Data Bank with the accession code 3IFV.

### Comparative size exclusion chromatography

*Hv*PCNA purified as described was fractionated on a 10/300 Superdex 200 (GE Healthcare) size exclusion column equilibrated in buffer containing 50 mM HEPES pH 7.0 and either 0.2 or 3.0 M KCl. Equivalent amounts of protein were incubated in the respective buffer for 4 hours prior to loading. The flow rate was maintained at 0.5 ml/min.

## Authors' contributions

JAW purified and crystallised *Hv*PCNA, carried out data collection and helped draft the manuscript. PC and SM cloned and expressed *Hv*PCNA. KAB initiated the study, carried out structure solution and refinement and drafted the manuscript.

## Supplementary Material

Additional file 1 **Figure S1 – Electrostatic surfaces of classical PCNAs. Figure S2 – Sodium ion binding at a crystal packing interface.** Table S1 – Interactions at the monomer-monomer interface. Table S2 – Amino acid usage in PCNAs. Table S3 – *B *factor analysis. Tables S4 and S5 – Na^+ ^coordination distances. Click here for file

Additional file 2 **Animation demonstrating the reduction in electrostatic potential in the *Hv*PCNA pore.** Click here for file

Additional file 3 **Animation demonstrating the electrostatic charge in the pore of a classical PCNA (*Af*PCNA).** Click here for file

## References

[B1] Barry ER, Bell SD (2006). DNA replication in the archaea. Microbiol Mol Biol Rev.

[B2] Soppa J (2006). From genomes to function: haloarchaea as model organisms. Microbiology.

[B3] Allers T, Ngo HP (2003). Genetic analysis of homologous recombination in Archaea: Haloferax volcanii as a model organism. Biochem Soc Trans.

[B4] Mullakhanbhai MF, Larsen H (1975). Halobacterium volcanii spec. nov., a Dead Sea halobacterium with a moderate salt requirement. Arch Microbiol.

[B5] Christian J, Waltho J (1962). Solute concentration within cells of halophlic and non-halophilic bacteria. Biochim Biophys Acta.

[B6] Britton KL, Baker PJ, Fisher M, Ruzheinikov S, Gilmour DJ, Bonete MJ, Ferrer J, Pire C, Esclapez J, Rice DW (2006). Analysis of protein solvent interactions in glucose dehydrogenase from the extreme halophile Haloferax mediterranei. Proc Natl Acad Sci USA.

[B7] Ebel C, Faou P, Kernel B, Zaccai G (1999). Relative role of anions and cations in the stabilization of halophilic malate dehydrogenase. Biochemistry.

[B8] Richard SB, Madern D, Garcin E, Zaccai G (2000). Halophilic adaptation: novel solvent protein interactions observed in the 2.9 and 2.6 A resolution structures of the wild type and a mutant of malate dehydrogenase from Haloarcula marismortui. Biochemistry.

[B9] Pieper U, Kapadia G, Mevarech M, Herzberg O (1998). Structural features of halophilicity derived from the crystal structure of dihydrofolate reductase from the Dead Sea halophilic archaeon, Haloferax volcanii. Structure.

[B10] Sivakumar N, Li N, Tang JW, Patel BK, Swaminathan K (2006). Crystal structure of AmyA lacks acidic surface and provide insights into protein stability at poly-extreme condition. FEBS Lett.

[B11] Kong XP, Onrust R, O'Donnell M, Kuriyan J (1992). Three-dimensional structure of the beta subunit of E. coli DNA polymerase III holoenzyme: a sliding DNA clamp. Cell.

[B12] Gulbis JM, Kelman Z, Hurwitz J, O'Donnell M, Kuriyan J (1996). Structure of the C-terminal region of p21(WAF1/CIP1) complexed with human PCNA. Cell.

[B13] Moarefi I, Jeruzalmi D, Turner J, O'Donnell M, Kuriyan J (2000). Crystal structure of the DNA polymerase processivity factor of T4 bacteriophage. J Mol Biol.

[B14] Matsumiya S, Ishino Y, Morikawa K (2001). Crystal structure of an archaeal DNA sliding clamp: proliferating cell nuclear antigen from Pyrococcus furiosus. Protein Sci.

[B15] Warbrick E (1998). PCNA binding through a conserved motif. Bioessays.

[B16] Dionne I, Nookala RK, Jackson SP, Doherty AJ, Bell SD (2003). A heterotrimeric PCNA in the hyperthermophilic archaeon Sulfolobus solfataricus. Mol Cell.

[B17] Bunting KA, Roe SM, Pearl LH (2003). Structural basis for recruitment of translesion DNA polymerase Pol IV/DinB to the beta-clamp. Embo J.

[B18] Chapados BR, Hosfield DJ, Han S, Qiu J, Yelent B, Shen B, Tainer JA (2004). Structural basis for FEN-1 substrate specificity and PCNA-mediated activation in DNA replication and repair. Cell.

[B19] Warbrick E (2000). The puzzle of PCNA's many partners. Bioessays.

[B20] Kontopidis G, Wu SY, Zheleva DI, Taylor P, McInnes C, Lane DP, Fischer PM, Walkinshaw MD (2005). Structural and biochemical studies of human proliferating cell nuclear antigen complexes provide a rationale for cyclin association and inhibitor design. Proc Natl Acad Sci USA.

[B21] Krishna TS, Kong XP, Gary S, Burgers PM, Kuriyan J (1994). Crystal structure of the eukaryotic DNA polymerase processivity factor PCNA. Cell.

[B22] Madern D, Ebel C, Zaccai G (2000). Halophilic adaptation of enzymes. Extremophiles.

[B23] Bieger B, Essen LO, Oesterhelt D (2003). Crystal structure of halophilic dodecin: a novel, dodecameric flavin binding protein from Halobacterium salinarum. Structure.

[B24] Krissinel E, Henrick K (2007). Inference of macromolecular assemblies from crystalline state. J Mol Biol.

[B25] Frolow F, Harel M, Sussman JL, Mevarech M, Shoham M (1996). Insights into protein adaptation to a saturated salt environment from the crystal structure of a halophilic 2Fe-2S ferredoxin. Nat Struct Biol.

[B26] Kelman Z, O'Donnell M (1995). Structural and functional similarities of prokaryotic and eukaryotic DNA polymerase sliding clamps. Nucleic Acids Res.

[B27] Besir H, Zeth K, Bracher A, Heider U, Ishibashi M, Tokunaga M, Oesterhelt D (2005). Structure of a halophilic nucleoside diphosphate kinase from Halobacterium salinarum. FEBS Lett.

[B28] Cann IK, Ishino S, Hayashi I, Komori K, Toh H, Morikawa K, Ishino Y (1999). Functional interactions of a homolog of proliferating cell nuclear antigen with DNA polymerases in Archaea. J Bacteriol.

[B29] Matsumiya S, Ishino S, Ishino Y, Morikawa K (2003). Intermolecular ion pairs maintain the toroidal structure of Pyrococcus furiosus PCNA. Protein Sci.

[B30] Lanyi JK (1974). Salt-dependent properties of proteins from extremely halophilic bacteria. Bacteriol Rev.

[B31] Mayanagi K, Kiyonari S, Saito M, Shirai T, Ishino Y, Morikawa K (2009). Mechanism of replication machinery assembly as revealed by the DNA ligase-PCNA-DNA complex architecture. Proc Natl Acad Sci USA.

[B32] Oakley AJ, Prosselkov P, Wijffels G, Beck JL, Wilce MC, Dixon NE (2003). Flexibility revealed by the 1.85 A crystal structure of the beta sliding-clamp subunit of Escherichia coli DNA polymerase III. Acta Crystallogr D Biol Crystallogr.

[B33] Meslet-Cladiere L, Norais C, Kuhn J, Briffotaux J, Sloostra JW, Ferrari E, Hubscher U, Flament D, Myllykallio H (2007). A novel proteomic approach identifies new interaction partners for proliferating cell nuclear antigen. J Mol Biol.

[B34] Fukuda K, Morioka H, Imajou S, Ikeda S, Ohtsuka E, Tsurimoto T (1995). Structure-function relationship of the eukaryotic DNA replication factor, proliferating cell nuclear antigen. J Biol Chem.

[B35] Fribourg S, Romier C, Werten S, Gangloff YG, Poterszman A, Moras D (2001). Dissecting the interaction network of multiprotein complexes by pairwise coexpression of subunits in E. coli. J Mol Biol.

[B36] Leslie AGW (1992). Recent changes to the MOSFLM package for processing film amd image plate data. Joint CCP4 + ESF-EAMCB Newsletter on Protein Crystallography.

[B37] (1994). The CCP4 suite: programs for protein crystallography. Acta Crystallogr D Biol Crystallogr.

[B38] Long F, Vagin AA, Young P, Murshudov GN (2008). BALBES: a molecular-replacement pipeline. Acta Crystallogr D Biol Crystallogr.

[B39] Vagin AA, Teplyakov A (1997). MOLREP: an Automated Program for Molecular Replacement. Journal of Applied Crystallography.

[B40] Murshudov GN, Vagin AA, Dodson EJ (1997). Refinement of macromolecular structures by the maximum-likelihood method. Acta Crystallogr D Biol Crystallogr.

[B41] Emsley P, Cowtan K (2004). Coot: model-building tools for molecular graphics. Acta Crystallogr D Biol Crystallogr.

[B42] DeLano WL (2008). The PyMol Molecular Graphics System.

[B43] Baker N, Sept D, Joseph S, Holst M, McCammon J (2001). Electrostatics of nanosystems: application to microtubules and the ribosome. Proc Natl Acad Sci USA.

[B44] Pettersen EF, Goddard TD, Huang CC, Couch GS, Greenblatt DM, Meng EC, Ferrin TE (2004). UCSF Chimera – a visualization system for exploratory research and analysis. J Comput Chem.

