# Supplementary material for: The crystal structure of Haloferax volcanii proliferating cell nuclear antigen reveals unique surface charge characteristics due to halophilic adaptation
Source: BMC Struct Biol. 2009 Aug 22;9:55. doi: 10.1186/1472-6807-9-55 (PMC2737543; doi:10.1186/1472-6807-9-55)
Supplement: Additional file 1 — Figure S1 – Electrostatic surfaces of classical PCNAs. Figure S2 – Sodium ion binding at a crystal packing interface. Table S1 – Interactions at the monomer-monomer interface. Table S2 – Amino acid usage in PCNAs. Table S3 – B factor analysis. Tables S4 and S5 – Na+ coordination distances. [file 1472-6807-9-55-S1.doc]

Supplementary information - Winter *et al*. The crystal structure of *Haloferax volcanii* proliferating cell nuclear antigen reveals unique surface charge characteristics due to halophilic adaptation.

**Figure S1.** Electrostatic surfaces of known PCNA structures demonstrating the conserved nature of the electropositive central pore, absent in *Hv*PCNA*.* A – *Hv*PCNA, B – *Af*PCNA, C – *Pf*PCNA, D – hPCNA and E – yPCNA*.* The electrostatic potential was calculated using the APBS package. The accessible surface area is coloured according to the calculated electrostatic potential from -10 k_B_T/e (red) to +10 k_B_T/e (blue).

A.
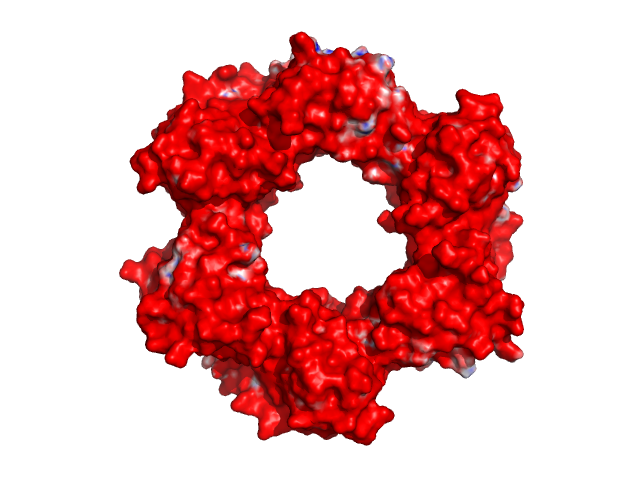


B.
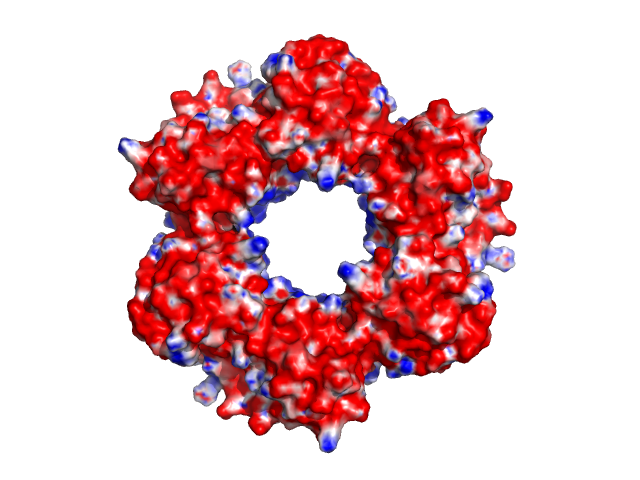
 C.
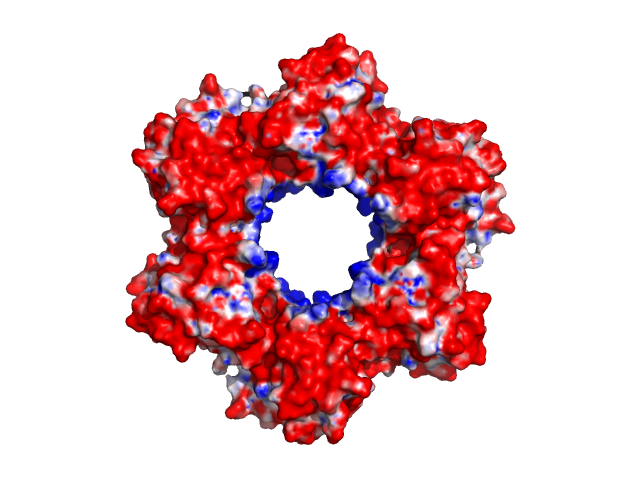


D.
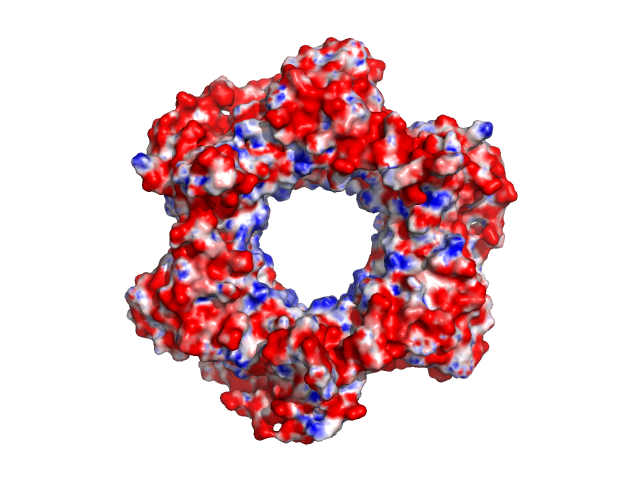
E.
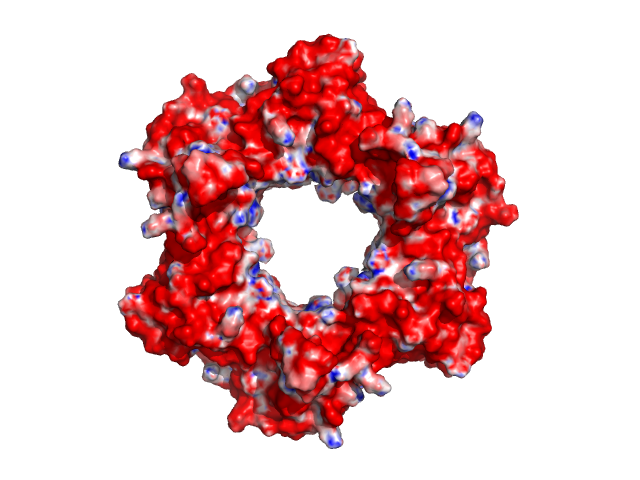


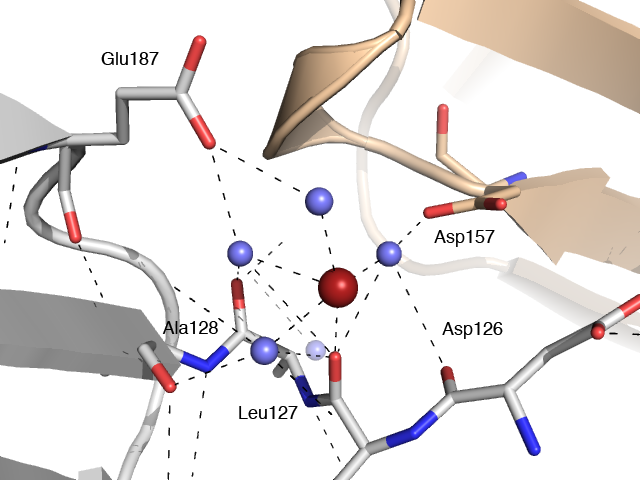


**Figure S2.** Sodium ion binding at a crystal packing interface. Interacting residues are shown in stick representation. Water molecules are shown in purple and sodium ions in red with hydrogen bonds indicated by dashed black lines. Chain C is shown in white, chain B in beige (symmetry related).


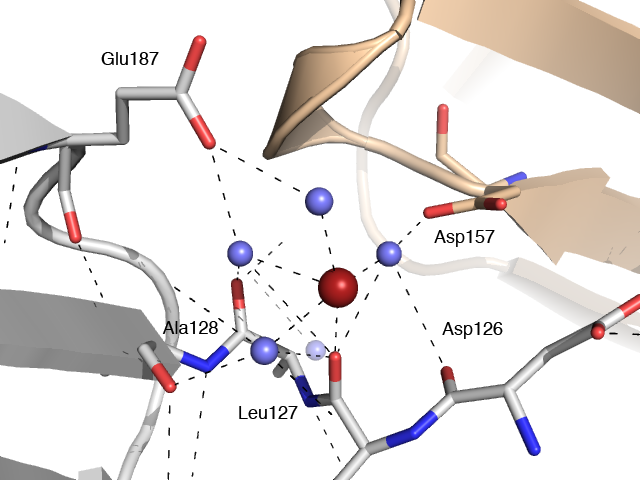
Glu187

|  | Average distance (Å) |
| --- | --- |
| Main chain |  |
| Gly103 O – Ser177 N | 3.00 |
| Ser105 N – Asp175 O | 2.76 |
| Ser105 O – Asp175 N | 2.74 |
| Thr107 N – Asp173 O | 2.78 |
| Thr107 O – Asp173 N | 3.07 |
| Side chain |  |
| Tyr106 OH – Asp172 OD2 | 3.23 |
| Ala109 N – Asp172 OD2 | 2.94 |
| Ion pair |  |
| Arg72 NH1 – Asp172 OD2 | 3.13 |

**Table S1** – Intermolecular bond distances at the monomer-monomer interface, averaged over the three ncs-related subunits.

| aa | *Hv* | *Af* | *Pf* | Yeast | Human | Swiss-Prot  average |
| --- | --- | --- | --- | --- | --- | --- |
| Ala | 11.3 | 7.8 | 6.4 | 5.0 | 7.3 | 8.1 |
| Arg | 4.5 | 4.9 | 3.6 | 3.1 | 3.1 | 5.5 |
| Asp | 11.3 | 7.8 | 6.8 | 10.5 | 6.9 | 5.4 |
| Glu | 8.5 | 10.2 | 13.3 | 7.4 | 8.8 | 6.7 |
| His | 2.4 | 1.6 | 0.4 | 0.8 | 1.1 | 2.3 |
| Ile | 8.1 | 10.2 | 7.6 | 9.3 | 5.4 | 5.9 |
| Leu | 12.1 | 7.8 | 10.4 | 12.4 | 1.1 | 9.7 |
| Lys | 2.8 | 7.8 | 8.4 | 7.0 | 6.1 | 5.9 |
| Phe | 2.0 | 4.9 | 4.8 | 6.6 | 3.1 | 3.9 |
| Ser | 6.1 | 6.5 | 4.8 | 9.3 | 9.6 | 6.7 |
| Thr | 4.9 | 4.1 | 5.6 | 4.7 | 4.6 | 5.4 |
| Trp | 0.0 | 0.0 | 0.0 | 0.0 | 0.4 | 1.1 |
| Val | 6.9 | 10.2 | 8.8 | 5.0 | 8.0 | 6.8 |
| Net charge | -31 | -13 | -20 | -20 | -17 |  |

**Table S2** - Comparison of % amino acid usage between known homotrimeric PCNA structures and overall for the UniProtKB/Swiss-Prot release July 2008. Also shown is the net charge, calculated assuming all histidines are neutral.

|  |  | *B* factor (Å^2^) |  |
| --- | --- | --- | --- |
| Molecule | Resolution (Å) | Protein (no. residues) | Water (no. molecules) in first hydration shell |
| *Hv*PCNA | 2.0 | A 28.45 (216)  B 28.53 (234)  C 21.30 (240) | 26.97 (81)  27.51 (85)  24.27 (141) |
|  |  |  | All waters |
| *Af*PCNA (1rwz) | 1.8 | 22.87 (244) | 39.19 (316) |
| *Pf*PCNA (1ge8) | 2.1 | 30.57 (238) | 37.09 (41) |
| hPCNA (1vym) | 2.3 | 51.16 (255) | 50.67 (47) |
| yPCNA (1plq) | 2.3 | 37.5 (258) | 43.95 (119) |

**Table S3** - *B* factor analysis of main chain atoms and water molecules, calculated using BAVERAGE. The hydration shells were assigned by WATERTIDY. hPCNA was averaged over 3 ncs related chains.

|  | ligand | A (Å) | B (Å) | C (Å) | Average (Å) |
| --- | --- | --- | --- | --- | --- |
| Na | Asp150 OD1 | 2.35 | 2.56 | 2.40 | 2.44 |
|  | Asp150 OD2 | 2.02 | 2.48 | 2.23 | 2.24 |
|  | water | 2.44 | 2.58 | 2.46 | 2.48 |
|  | water | 2.09 | 2.07 | 2.19 | 2.12 |
|  | water | 2.67 | 2.49 | 2.46 | 2.54 |
|  | water | 2.82 | 2.16 | 2.53 | 2.51 |
|  | water | 2.75 | 2.05 | 2.10 | 2.30 |
| Na | Asp146 OD1 | 2.42 | 2.56 | 2.28 | 2.42 |
|  | Asp146 O | 2.30 | 2.60 | 2.36 | 2.42 |
|  | water | 2.65 | 2.67 | 2.52 | 2.61 |
|  | Asp150 OD1 | 2.35 | 2.10 | 2.26 | 2.24 |
|  | water | 2.45 | 2.41 | Not present | 2.43 |
|  | Ser149 O | 2.36 | 2.43 | 3.48 | 2.42 |

**Table S4** – Na+ coordination at the double sodium site, showing bond lengths (Å) for each chain (A/B/C) and the average value. Water molecules were not assigned where density was insufficient.

| Ion | Distance (Å)  Chain A/B/C | Partner | Distance (Å)  Chain A/B/C | Partner |
| --- | --- | --- | --- | --- |
| Na | 2.61/2.55/2.42  2.53 | Leu 127 (C) O |  |  |
|  | 1.75/2.03/2.10  1.96 | HOH | 2.72/2.56/2.23  2.50  2.68/2.88/2.95  2.84  3.07/3.00/2.79  2.95 | Asp 157 (sym) OD1  Asp 157 (sym) OD2  Leu 127 O |
|  | 2.74/2.60/2.51  2.62 | HOH | 2.42/2.75/2.86  2.68  3.10/2.64/2.89  2.88 | Ala 129 O  Glu 126 OE2 |
|  | 2.13/2.48  2.31 | HOH * | 2.57/3.01  2.79 | Glu 187 OE1 |
|  | 2.66 | HOH &* | 3.02  2.58 | Glu 187 OE1  Ala 128 O |

**Table S5** – Na+ binding at a crystal packing interface. Distances are shown for each subunit and the average value is indicated in bold. Water molecules absent in the A (&) and B (*) subunits are indicated.
